# Editorial: Psychometrics in psychiatry 2022: psychological therapy and psychosomatics

**DOI:** 10.3389/fpsyt.2023.1295716

**Published:** 2023-10-18

**Authors:** Pasquale Scognamiglio, Phillip J. Tully, Mohsen Khosravi, Francesco Monaco

**Affiliations:** ^1^Department of Mental Health, ASL Napoli 3 Sud, Torre del Greco, Italy; ^2^Biopsychosocial Health and Wellbeing Lab, School of Psychology, The University of New England, Armidale, NSW, Australia; ^3^Department of Psychiatry, School of Medicine, Zahedan University of Medical Sciences, Zahedan, Iran; ^4^Health Promotion Research Center, Zahedan University of Medical Sciences, Zahedan, Iran; ^5^Department of Mental Health, Azienda Sanitaria Locale Salerno, Salerno, Italy; ^6^European Biomedical Research Institute of Salerno, Salerno, Italy

**Keywords:** psychometrics, psychosomatics, psychological therapy, alexithymia, metacognition, questionnaire, scales

The importance of evidence-based assessment and measurement in mental healthcare has become increasingly recognized in recent decades. However, psychiatry has long grappled with challenges in quantifying subjective psychological constructs and patient experiences into objective metrics (1). Unlike other medical specialties that can rely on quantifiable biomarkers (2), psychiatry relies heavily on patient self-reports, clinical evaluations, and rater-based scales. The field of psychiatry has long struggled with the issue of accurately and reliably measuring and assessing mental health constructs and disorders. The subjective nature of psychiatric symptoms and presentations contributes to challenges in quantitative measurement and in evaluating the efficacy of interventions. However, the importance of evidence-based assessment and treatment in mental healthcare cannot be understated. This makes scientifically rigorous assessment and measurement difficult. Historically, psychiatric diagnosis and treatment depended predominantly on clinical intuition rather than empirical data (3, 4). However, the rise of evidence-based medicine, and movements pushing for more reliable and valid psychiatric assessment, led to growing appreciation of the need for quantitative metrics and psychometrics. With the recent replication crisis highlighting issues in reproducibility of psychiatric research, improving measurement methodology has become even more critical (5).

Modern efforts to strengthen psychometrics in psychiatry date back to the post-World War II (6) era with the publication of the first Diagnostic and Statistical Manual of Mental Disorders (DSM) in 1952. The DSM (7) introduced standardized criteria for diagnosis along with a multiaxial system for broader assessment on factors like personality and intelligence. Quantitative rating scales for various disorders also emerged during this period, though uptake was limited. The 1980 DSM-III revolutionized psychiatric classification by establishing descriptive diagnostic criteria grounded in clinical consensus for the first time. This shift toward explicit, reliable diagnoses laid the foundation for the current emphasis on measurement-based care. The evolution of structured and semi-structured interviews, symptom rating scales, and self-report questionnaires accelerated in the 1980s and 90s. These tools enabled quantification of patient presentation and treatment effects (8).

Recent years have seen increasing diversity and sophistication of psychometric instruments for mental health assessment. There has also been greater recognition of the importance of measurement-based care and quantitative data in research and clinical practice. Routine outcome monitoring using validated rating scales has become a standard. Modern statistical methods and technological advances have further enhanced psychometric techniques in psychiatry. Item response theory and other new methodologies allow refinement of existing scales. Ecological momentary assessment uses digital tools to sample real-time patient experiences (9). Machine learning and artificial intelligence can help customize assessments and predict outcomes (10).

However, significant limitations around validity, reliability, and standardization of measures persisted and persists in psychiatry (11). There are still no established biomarkers or quantitative tests compared to other medical specialties even with the Research Domain Criteria (RDoC) framework (2). Additional research and development focused on psychometric testing is critical for continued progress.

This Research Topic aimed to advance efforts to strengthen psychometrics in psychiatry by showcasing innovative work on developing and applying quantitative measures, assessments, and methodologies, particularly for psychological therapy and psychosomatic conditions.

The study by Larionow et al. validated a Polish version of the Perth Alexithymia Questionnaire (PAQ), which assesses difficulty identifying and describing feelings and externally-oriented thinking. The PAQ's 5-factor structure was confirmed via confirmatory factor analysis in a large Polish adult sample. The PAQ demonstrated good convergent validity with another alexithymia measure and markers of psychological distress. Alexithymia levels were higher in younger vs. older adults. Females showed greater difficulty appraising negative vs. positive emotions compared to males. Overall, results supported the reliability and validity of the Polish PAQ as a comprehensive, multidimensional measure of alexithymia across valences, facilitating assessment and research among Polish speakers.

The paper by Martiadis et al. reviews psychometric tools for assessing metacognition in schizophrenia. Metacognition refers to thinking about thinking and is impaired in schizophrenia. Instruments discussed include semi-structured interviews like the Indiana Psychiatric Illness Interview that elicit narratives analyzed with the Metacognition Assessment Scale, self-reports like the Metacognitions Questionnaire, and clinician-rated tools. These assess metacognitive domains including understanding one's own mind, others' minds, and mastery or coping ability. Choosing appropriate tools depends on population, purpose, time, and training requirements. Further research on instruments to track metacognitive gains from therapies is needed. Overall, validated measures enable quantification of this complex cognitive capacity relevant to schizophrenia symptoms and functioning.

The research by de Beurs et al. established norms and T-scores for common mental health screeners in a large sample from Suriname. Measures assessed alcohol use (AUDIT), depression (CES-D), and anxiety (GAD-7, ACQ, BSQ). Compared to other countries, Surinamese people had lower problematic drinking but higher anxiety. Women scored higher on depression/anxiety while men had more alcohol issues. Norm tables provide score interpretations. Converting raw scores to normalized T-scores enables direct comparison across measures on a common metric. *T*-score thresholds aligned with standard cut-offs for disorder severity. Overall, locally-appropriate norms and *T*-score conversion aid interpretation, comparison, and clinical use of mental health screening scales in Suriname.

Xiao et al. developed and validated the Hospitalized Patients' Expectations for Treatment Scale (HOPE-P) to assess inpatients' expectations in general hospitals. Two hundred and ten patients in China completed the 9-item HOPE-P measuring doctor-patient communication, treatment outcomes, and disease management. Exploratory and confirmatory factor analyses supported a 2-factor structure (communication and outcomes) with strong model fit. The HOPE-P demonstrated good reliability and convergent validity. Patients reported high treatment expectations, especially regarding communication and outcomes. The HOPE-P provides a brief, reliable tool to quantify patient expectations, inform clinical care, and potentially improve patient safety management in hospitals. Further research on its clinical utility is warranted.

The work by Dębska et al. presented a study conducted to assess the psychometric properties of the Polish translation of the Transplant Effects Questionnaire (TxEQ-PL). The questionnaire measures emotional reactions of organ transplant recipients, including worry about the transplant, guilt toward the donor, disclosure of transplantation, adherence to medical treatment, and responsibility to the donor or medical staff. The study involved 84 kidney transplant patients, and the results indicated satisfactory internal consistency of the TxEQ-PL. The questionnaire showed significant relationships with factors such as optimism, depression, anxiety, and quality of life. Overall, the TxEQ-PL proved to be a useful tool for assessing emotional reactions to organ transplantation in Poland.

Taken together, the diverse array of papers in this Research Topic underscore the vital role that psychometrics and quantitative analysis play in 21st century psychiatry. The contributed articles highlight exciting new directions in elevating measurement rigor in mental healthcare. By aggregating cutting-edge research focused on quantifying subjective psychological constructs, this Research Topic provides an important forum for advancing evidence-based assessment in psychiatry. The field continues to move forward in translating the intricacies of mental illness into empirical data that can guide diagnosis and effective intervention.

## Author contributions

PS: Writing—original draft, Writing—review and editing. PT: Writing—review and editing. MK: Writing—review and editing. FM: Writing—review and editing.
